# The effect of the Baduanjin exercise on COVID-19-related anxiety, psychological well-being and lower back pain of college students during the pandemic

**DOI:** 10.1186/s13102-022-00493-3

**Published:** 2022-06-08

**Authors:** Keqiang Li, Tamara Walczak-Kozłowska, Mariusz Lipowski, Jianye Li, Daniel Krokosz, Yuying Su, Hongli Yu, Hongying Fan

**Affiliations:** 1 Gdansk University of Physical Education and Sport, Gdańsk, Poland; 2 Division of Neuropsychology, Department of the Social Sciences, Institute of Psychology, University of Gdańsk, Gdańsk, Poland; 3 Physical Education College, Bohai University, Jinzhou, China; 4 School of Psychology, Beijing Sport University, Beijing, China

**Keywords:** Qigong, Baduanjin exercise, College student, COVID-19-related anxiety, Mental health, The prevalence of low back pain, Pandemic

## Abstract

**Background:**

This study aimed to examine the effect of Baduanjin exercise on COVID-19-related anxiety, psychological well-being, and the lower back pain of college students during the coronavirus pandemic in China.

**Setting:**

The study was carried out in a temporary experimental center of four universities in Wenzhou city in Zhejiang Province, China.

**Population:**

387 participants who were college students were allocated to two groups: the Baduanjin exercise group(BEG, n = 195); and the Control group(CG,n = 192).

**Methods:**

In this randomized controlled trial,387 participants who were college students were randomly allocated in a 1:1 ratio to 12-week Baduanjin exercise group (BEG, n = 195)and 12-week Control group(CG,n = 192).CAS(Coronavirus Anxiety Scale), PWBS(Psychological Well-being Scale),NMQ( Nordic Musculoskeletal Questionnaire), was used to assess COVID-19-related anxiety, psychological well-being, and lower back pain at second times ( before and after the intervention). The paired t-test and an independent t-test (with a 95% confidence interval) was used to compare the outcome variables of the two groups.

**Results:**

Within-group comparison, there was no significant difference in the control group before and after the intervention. In contrast, the Baduanjin group had a significant improvement before and after the intervention. Between-group comparison, the Baduanjin group had a significant difference from the control group. The intervention effect on the Baduanjin exercise group was remarkably better than that of the control group (p < 0.05). Participants in the Baduanjin group significantly improved the corvid-19-related anxiety score decreased from ( 5.22 ± 0.45 to 5.07 ± 0.27, p < 0.05). The total psychological well-being score increased from (70.11 ± 8.65 to 84.12 ± 7.38,p < 0.05) and the prevalence of low back pain decreased from (22.45 ± 1.67 to 18.35 ± 1.05, p < 0.05) among college students.

**Conclusion:**

During the pandemic, the Baduanjin exercise contributes to the reduction of the perceived anxiety related to COVID-19, decreases the prevalence of the lower back pain, and improves the psychological well-being of college students.

***Trial registration*:**

Clinicaltrials.gov, NCT04432038. Registered on June 16, 2020.

## Background

Since the beginning of the COVID-19 pandemic until May 2021, numerous changes and restrictions have been introduced by countries that have significantly influenced the lifestyle of the inhabitants. People's traditional daily life and physical activities in China were highly affected by the government’s policy as well as by the trajectory of changes in the pandemic situation (e.g. changes in the spread of COVID-19 virus, periods of increased mortality and morbidity). Meanwhile, the pandemic has made Chinese universities lockdown, and college students have to study online courses at home. Long-term online courses have increased students' sedentary time, while pandemics have reduced daily exercise and social time [1]. Long-term sedentary behavior resulting from COVID-19’s isolation policy may have physical and psychological effects on college students [2–6], Those effects can lead to increase of the severity and prevalence of chronic diseases in different groups [7–17].

Numerous studies have shown that sedentary behavior has become a leading global public health problem, and as we all know this behavior is an important risk factor of several diseases, and even loss of life. In the sedentary behavior, the neck and lumbar spine and other body parts stay dormant for a long time. Students typically spend many hours seated on non-ergonomic chairs and assuming incorrect postures to carry out their curricular activities, leading to a general musculoskeletal overload [18], especially at the neck and low back [19]. Haroon et al. [20] reported university students use laptops for more than three hours per day as a risk factor for neck pain. This leads to an abnormal body position, causing pain in the waist, abdomen and back, in addition to decreased muscle strength. Sedentary behavior is one of the main risk factors for various chronic noncommunicable diseases, such as spondylosis, periarthritis of shoulder, nonspecific low back pain and many others [21, 22]. It also increases the severity of perceived stress, and reduces people's quality of life and psychological well-being. Relevant research shows that chronic pain increases patient Perceived stress when our body perceives stress [23, 24]. Approximately 15 min after the onset of stress, cortisol levels rise systemically and remain elevated for several hours [25, 26]. However, although a stress-induced increase in cortisol secretion is adaptive in the short-term, excessive or prolonged cortisol secretion may have crippling effects, both physically and psychologically [24, 27, 28].and thus should be recognized as an additional risk factor during the pandemic when people generally report greater fear and more worries [29, 30]. There is growing evidence pointing that the COVID-19 pandemic contributes to the increased number of cases of mental health problems, anxiety, and depression [31–34].

Baduanjin, in terms of its effect as an exercise intervention, has been recognized in many international studies [35]. As a traditional Chinese mind-body aerobic exercise, Qigong is based on Taoist philosophy and traditional Chinese medicine theories [36]. Qigong is a combination of postures, meditation, and movements designed to improve holistic health and to facilitate mind-body integration [36, 37]. It consists of eight independent, simple, subtle, and smooth movements [38]. Although the potential effectiveness of each movement may be different, the overall Baduanjin exercise has been demonstrated to have a good effect on body and mind [38]. Baduanjin exercise can enhance Qi function through the whole exercise of body posture, movement, breathing, and meditation—that is, to draw upon natural forces to optimize and balance energy within, through the purposeful coordination of body, breath, and mind [39]. With the combination of self-awareness with self-correction of the posture and movement of the body, the flow of breath, and stilling of the mind, Baduanjin exercise is thought to comprise a state which activates the natural self-regulation capacity, stimulates the balanced release of endogenous neurohormones, and a wide array of natural health recovery mechanisms [40].

For instance, the Baduanjin exercise has been recognized as an effective way of alleviating the lower back pain (even its chronic condition) caused by lumbar disc herniation [41, 42]. It improves the strength and flexibility of the neck, shoulder, and back. Thus, it helps reducing the occurrence and development of diseases related to the above-mentioned parts of the body [43, 44]. The positive effect of the Baduanjin exercise on mental health was previously observed in cohorts of college students, as well as middle-aged and older people [45–47]. Beside, studies show that Baduanjin played an obvious role in adjuvant therapy and postoperative rehabilitation during COVID-19 epidemic [48–50].

However, little is known about the effect of the Baduanjin exercise on the prevalence of lower back pain, COVID-19-related anxiety and psychological well-being in college students during the pandemic. Thus, this article aims to explore the effects of Baduanjin intervention on physical and mental health of people during the pandemic, together with providing theoretical and practical references for healthy lifestyles for sedentary groups.

## Materials and methods

### Design

**Fig. 1:**
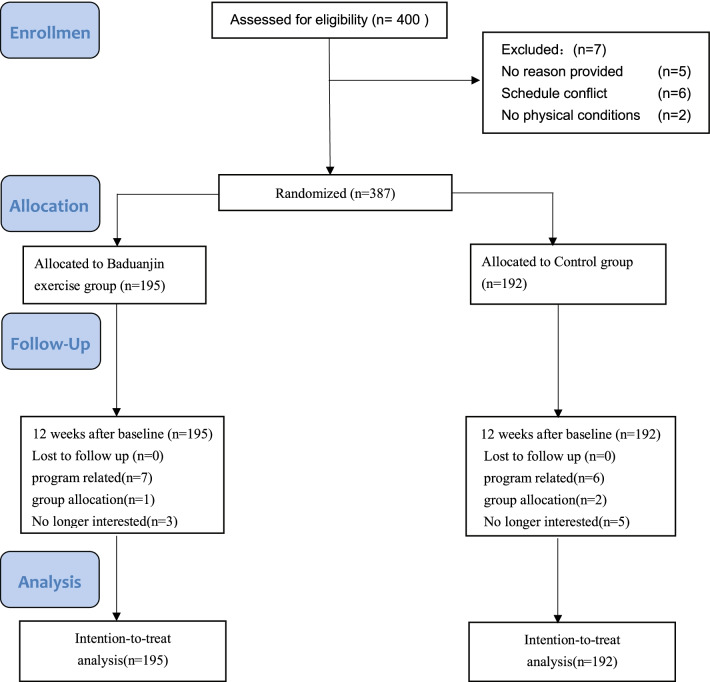
Study flow diagram of the progress through the phases of the experiment

The purpose of the study was to examine whether Baduanjin exercises can decrease COVID-19-related anxiety, decrease the prevalence of lower back pain, and increase psychological well-being in College Students during the pandemic. The protocol of this study was approved by the Ethics Board for Research Projects at the Institute of Psychology, University of Gdansk, Poland (decision no. 33/2020). Figure 1 presents the stages of the study regarding also the flow of the respondents.

### Participants

From September 2020 to December 2020, four universities students were randomly selected from Wenzhou City in Zhejiang Province, China. According to the inclusion criteria, the participants were between 20 and 30 years old. Furthermore, they must have worked, studied, or lived in a selected school for more than two months in the past year and needed to be able to participate in online and offline activities as well as answer questions from the surveys. Eventually, a total of 387 participants were qualified for the study.

### The intervention

#### 2.3.1 Randomization and allocation

387 participants who fulfilled the eligibility criteria were allocated randomly to two intervention-based groups; the Baduanjin exercise group (BE; n = 195) or the Control group (CG; n = 192). To ensure blinding, an independent researcher who was not a part of this study performed the randomized allocation. A 1:1 simple randomization technique was employed. A unique, computer-generated random code was assigned for each participant via SPSS (version 26.0, Armonk, New York, USA) statistical software. The allocation was concealed in sealed opaque envelopes, which were provided to researchers before applying the assigned interventions. All study personnel and participants were blinded to treatment assignment for the trial duration. The experimental intervention time for the two groups was 12 weeks.

#### Baduanjin exercise group

In this group, the participants were performing the Baduanjin exercises for 12 weeks. They were guided by a professional Qigong Baduanjin coach in the first week and then began a formal 11-week intervention period of Baduanjin exercises after mastering moving and breathing methods. During this intervention period, the participants were exercising independently, and each week they have been doing the Baduanjin exercises no fewer than five times for a total duration of 45 min per session [51]. They were required to wear sportswear and sports shoes during these exercises. Participants performed a warm-up exercise for approx. 5–10 min before the practice, with formal exercises beginning after 3–5 min of Qi practicing. Stretching exercises for 5 min after the exercise were also performed by the participants. The coach guided subjects through a group exercise once a week (during the formal 11-week intervention period). Participants need to provide instant feedback which they had encountered in their daily exercises to the doctor and coach at any time.

#### Control group

To examine the validity of the pre and post-test learning health knowledge, participants needed to join a WeChat group to finish the test. Participants were instructed to learn health knowledge on the Internet independently during the learning process. They were asked to be learning for 12 consecutive weeks. Each week, they had to learn no fewer than 5 times and learn at least 30 min every time. To ensure that participants learn health knowledge independently, each participant was asked to check-in before and after learning in a WeChat group. And participants had to complete the quiz after learning the health knowledge course each time. Management staff undertook a return visit to the control group every other week and showed each course's quiz results.

### Measurements

#### Nordic musculoskeletal questionnaire (NMQ)

The Nordic Musculoskeletal Questionnaire was applied to measure the musculoskeletal pain [52–54]. Nordic Musculoskeletal Questionnaire (NMQ) were collected [55]. NMQ is valid, reliable and responsive [56, 57]. Both English and Chinese versions of the NMQ have been used in several studies for the analysis of musculoskeletal symptoms in an occupational health context [58].This questionnaire addresses three main parts. The first part is about any trouble (such as pain, ache, discomfort and numbness) felt by the respondent in the last 12 months. The second part asks the same question but for the last seven days. The final part is about the disability caused by the trouble in the last 12 months. In each part, the data collected were about different anatomical areas: neck, upper back, lower back, shoulders, elbows, wrists/hands, hips/thighs/buttocks, knees and ankle/feet. The responses were recorded in the form of binary options (“yes” and “no”).Severe pain was assessed using the Numerical Pain Rating Scale [59], which classifies a score of eight or higher as severe pain. Severe pain was classified according to the criteria of Boonstra et al. (2016), which considers: no pain (score = 0), mild pain (≤ 5), moderate pain (6 and 7), and severe pain (≥ 8) [60].Previous studies have demonstrated that NMQ is well-validated [35].The internal consistency of the Nordic Musculoskeletal Questionnaire in our study was α = 0.93.

#### The coronavirus anxiety scale (CAS)

For the research purposes, we used the Coronavirus Anxiety Scale (CAS) developed by [61] which consists of 5 items developed based on the psychological descriptions of fear and anxiety symptoms [38, 62–64]. The five items of the CAS (Lee, 2020) were presented to the participants: dizziness, sleep disturbances, tonic immobility, appetite loss, and abdominal distress. Participants had to rate them on the same 5-point scale as in the original version (How often have you experienced any of the following in the past 2 weeks? 0 = not at all; 1 = rare, less than a day or two; 2 = several days; 3 = more than 7 days; 4 = nearly every day over the last 2 weeks). The total score is calculated by adding the scores for each of the responses [61]. The internal consistency of the Coronavirus Anxiety Scale CAS scale in our study was α = 0.793.

#### Psychological well-being scale (PWBS)

This questionnaire was originally designed and proposed by Ryff [65], yet we used the version developed by Ryff and Almeida [66] for this study, which is more in line with the current pandemic situation. After being quoted by many experts and scholars [67–69], it’s reliability was scored as being high. PWBS is a measurement tool with a multi-dimensional structure, this scale has six subscales. They are principal elements of psychological well-being. These subscales are autonomy, personal growth, environmental mastery, purpose in life, positive relations with others, and self-acceptance. Each subscale entails three items rated on a 6-point Likert scale (from strongly disagree = 1 to strongly agree = 6). Eight-item this scale are reverse scored. This scale has a score range of 18–108, with a higher score indicating better PWB.The internal consistency of the PWBS total scale in our study was α = 0.78.

### Statistical analyses

IBM SPSS Statistics, Version 26.0 (IBM, Armonk, New York, USA) was used for statistical analysis. A one-way analysis of variance and chi-square tests were used to analyze baseline demographic characteristics between two groups. After analyzing normal distribution with the Kolmogorov-Smirnoff (K-S) test, the descriptive characteristics of variables were expressed using means and standard deviations (SD). The paired t-test (t-test for dependent variables) was used to verify the group's changes before and after the intervention. An independent sample t-test (t-test for independent variables with a 95% confidence interval) was used to compare the two groups' mean values after the intervention. The tests were conducted to examine the effect of Baduanjin exercise on COVID-19-related anxiety, psychological well-being, and the prevalence of lower back pain of college students. P-value of 0.05 has been adopted for the standard evaluation of significant differences, and *p* = 0.01—for noticeable significant differences. The correlation analysis was used to analyze the correlation between the prevalence of lower back pain, changes in psychological well-being, and improvement of COVID-19-related anxiety.

## Results

### Descriptive statistics of sociodemographic information of Baduanjin exercise group and controls

A total of 387 participants were in this study, and most of them were College/Undergraduate students (57.62%). Men covered 50.9%. There were no significant differences between the Baduanjin exercise group and the control group regarding gender, age, education level, marital status, smoking history, drinking history, Sedentary time, Frequency of participation in exercise (*p* > 0.05). More detailed statistics of the participants’ characteristics are presented in Table 1.

**Table 1:** Descriptive statistics of sociodemographic information of Baduanjin exercise group and controls; n = 387

Variable		BEG (n = 195)	CG (n = 192)	*X*^2^	*p*
Age (years)		24 ± 4	23 ± 3	− 0.32	0.964
Gender (M/F)	Men	106 (54.3%)	91 (47.3%)		0.716
	Female	89 (45.7%)	101 (52.7%)		
Education	College/Undergraduate	114 (58.4%)	109 (56.7%)	1.493	0.439
	Postgraduate Student	49 (25.25%)	61(31.9%)		
	Doctoral candidate	20(10.4%)	22(11.4%)		
Marital status	Married	19 (9.7%)	13 (6.7%)	0.719	0.35
	Other	176 (90.3%)	179 (93.3%)		
Smoking status	Never Yes Ever smoking	104 (53.3%) 65 (33.3%) 26 (13.4%)	99 (51.56%) 70 (36.4%) 23 (12.04%)	0.819	0.631
Drinking status	Never Yes Ever drinking	106 (54.35%) 77 (39.4%) 12 (6.25%)	109 (56.7%) 69 (35.9%) 14 (7.4%)	0.496	0.774
Sedentary time (hour)	≤ 5 5–9 10 ≥	40 (20.5%) 58 (29.8%) 97 (49.7%)	37 (19.2%) 63 (32.9%) 92 (47.9%)	1.33	0.628
Frequency of participation in exercise (times / week)	≤ 3 3–5 5 ≥	83 (42.5%) 70 (35.8%) 42 (21.7%)	78 (40.6%) 67 (34.8%) 47 (24.6%)	− 0.25	0.356

In the case of variables with a quantitative measurement scale, the data was presented using mean ± standard deviation

### The musculoskeletal pain among the participants of the study

**Fig. 2:**
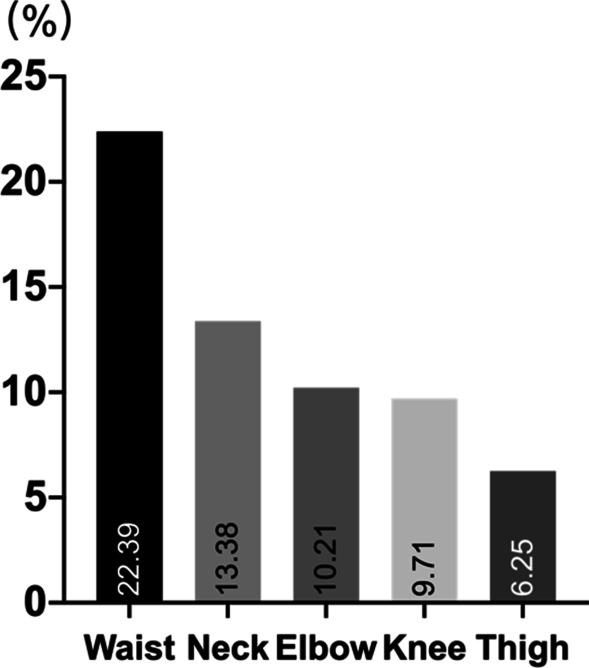
The musculoskeletal pain among the participants of the study; n = 387

Before we started analyzing between-group and within-group differences, we decided to find out which part of the body college students most commonly experience pain. The analysis of scores obtained in the Nordic Musculoskeletal Questionnaire (NMQ)[35] before the intervention (at baseline) revealed that among 387 of participants, the highest prevalence of pain was in the waist (22.39%), and the lowest prevalence—in the thigh (6.25%). The details are presented in Fig. 2.

### Nordic musculoskeletal questionnaire (NMQ): before and after the intervention

**Fig. 3:**
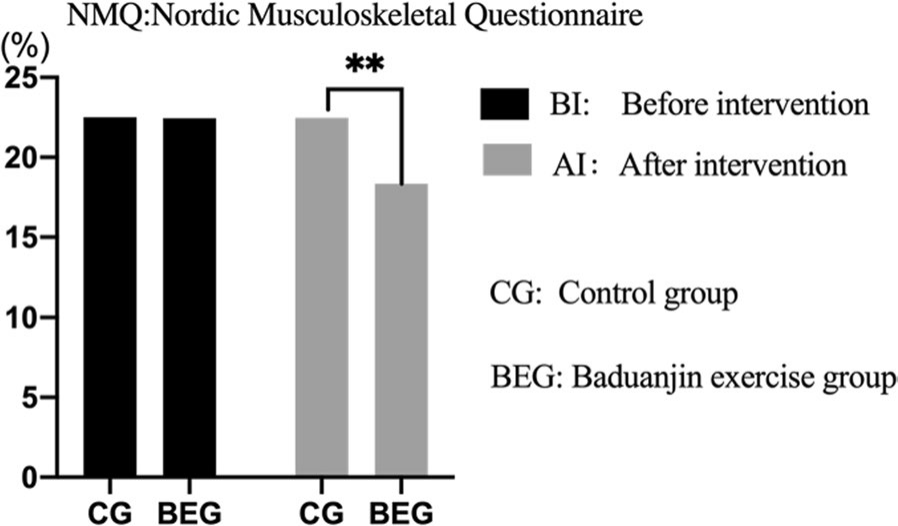
NMQ: before and after intervention between the two groups: control group vs. Baduanjin exercise group. p* < 0.05; p** < 0.01

Before the intervention, there were no significant differences in scores obtained in the Nordic Musculoskeletal Questionnaire (NMQ) between BEG and CG (*p* > 0.05), and after the intervention, BEG group scored significantly lower than CG group in NMQ (*p* > 0.05). There was significant decrease in scores obtained in NMQ between the two assessments in the BEG group, yet the difference between the two measurements in the CG group was insignificant (see Table 2 and Fig. 3 for the details).

**Table 2:** Nordic Musculoskeletal Questionnaire (NMQ): scores before and after intervention in the two groups (BEG vs. CG); n = 387

Group	Before intervention	After intervention	*p*^2^
CG(n = 192)	22.51 ± 1.81	22.47 ± 1.46	> 0.05
BEG (n = 195)	22.45 ± 1.67	18.35 ± 1.05**	< 0.05
*p*^1^	> 0.05	< 0.05	

*CG* Control group, *BEG* Baduanjin exercise group

^1^ Between-group difference

^2^Within-group difference

^*^p < 0.05

^**^p < 0.01

### Coronavirus anxiety scale (CAS): before and after the intervention

**Fig. 4:**
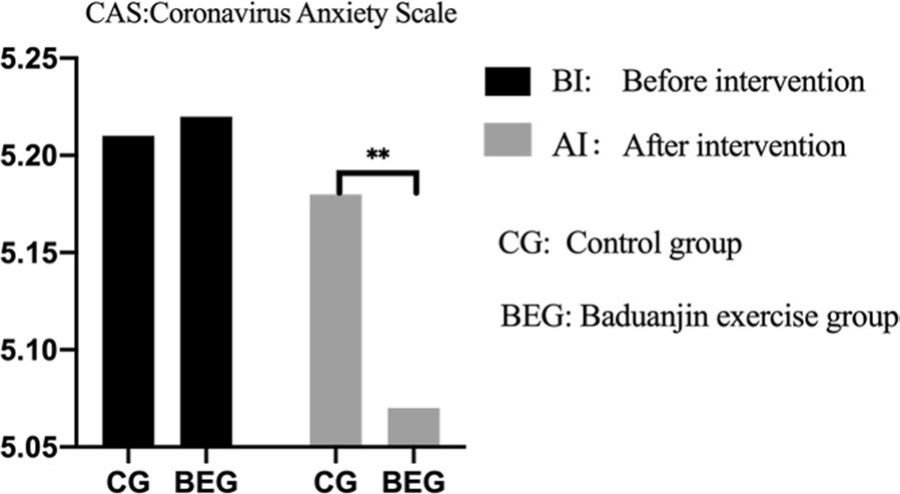
CAS: before and after intervention between the two groups: control group vs Baduanjin exercise group; *p* * < 0.05; *p* ** < 0.01

Before the intervention, there was no significant difference between BEG and CG in scores obtained in the CAS (*p* > 0.05), however, such a between-group difference was found after the intervention (*p* < 0.05). Moreover, in both—the BEG and CG, the scores obtained in CAS decreased in exercise time (BEG: *p* < 0.01; CG: *p* < 0.05). The Baduanjin exercise group was significantly lower than the control group (*p* < 0.05). Table 3 and Fig. 4 provides more details.

**Table 3:** Coronavirus Anxiety Scale (CAS) before and after intervention between the two groups (BEG vs. CG); n = 387

Group	Before intervention	After intervention	*p*^2^
CG(n = 192)	5.21 ± 0.67	5.18 ± 0.78	< 0.05
BEG (n = 195)	5.22 ± 0.45	5.07 ± 0.27**	< 0.01
*p*^1^	> 0.05	< 0.05	

Control group; *BEG* Baduanjin exercise group

*p*^1^: Between-group difference

*p*^**2**^: Within-group difference

^*^p < 0.05

^**^p < 0.01

### Psychological well-being (PWBS): before and after intervention

**Fig. 5:**
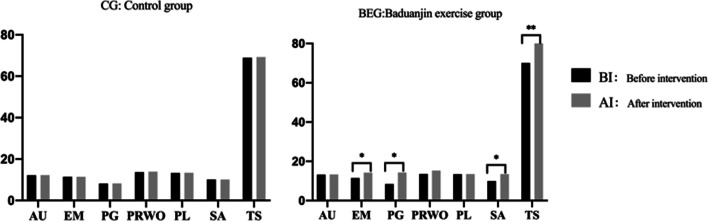
PWBS: before and after intervention between the two groups: control group vs Baduanjin exercise group; *p < 0.05; **p < 0.01

Before the intervention, there were no significant differences in the PWBS scores between BEG and CG (*p* > 0.05). However, groups differed significantly after the intervention (p < 0.05) with BEG group scoring significantly better in environmental mastery, personal growth, self-acceptance, and PWBS total score. Significant improvement between the two assessments was observed only in the BEG group: participants received better scores in such PWBS’s subscales as environmental mastery, personal growth, positive relations with others, self-acceptance, and in the PWBS total score (see Table 4 and Fig. 5 for the details).

**Table 4:** Psychological Well-Being (PWBS) before and after intervention between the two groups (BEG vs. CG); n = 387

Group		AU	EM	PG	PRWO	PL	SA	TS
CG (n = 192)	Before intervention	12.19 ± 2.12	11.40 ± 1.68	8.15 ± 2.21	13.73 ± 2.15	13.33 ± 2.38	10.12 ± 1.67	68.92 ± 6.72
	After intervention	12.22 ± 1.81	11.43 ± 2.01	8.23 ± 1.89	13.88 ± 2.46	13.39 ± 2.63	10.07 ± 1.35	69.22 ± 5.34
BEG (n = 195)	Before intervention	13.21 ± 2.06	11.48 ± 2.19	8.45 ± 2.33	13.54 ± 2.47	13.46 ± 2.24	9.97 ± 1.38	70.11 ± 8.65
	After intervention	13.28 ± 2.37	14.23 ± 2.04*	14.31 ± 2.43*	14.32 ± 1.78	13.51 ± 2.27	13.47 ± 2.65*	84.12 ± 7.38**

AU: Autonomy; EM: Environmental Mastery; PG: Personal Growth; PRWO: Positive Relations with Others; PL: Purpose in Life; SA: Self-Acceptance; TS: PWBS total score; CG: Control group; BEG: Baduanjin exercise group

*p < 0.05; **p < 0.01

### Correlation between the prevalence of low back pain, changes of psychological well-being, and improvement of COVID-19 related anxiety

**Fig. 6:**
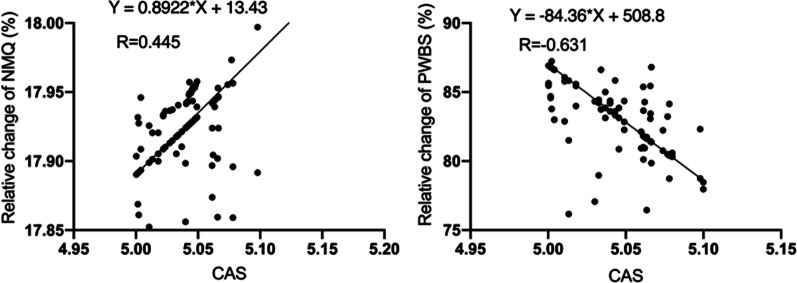
Correlations between changes in the prevalence of low back pain, psychological well-being and Coronavirus-related anxiety

After 12 weeks of intervention, COVID-19-related anxiety, the prevalence of low back pain and psychological well-being in participants in the two groups have improved in comparison to the assessment before the intervention. Partial correlation analysis was used to analyze the correlation between the prevalence of low back pain, changes in psychological well-being, and improvement of COVID-19 related anxiety. The results (Table 5) show that when controlling for age and sex, there is a significant positive correlation between the changes in the prevalence of low back pain (NMQ) and the COVID-19-related anxiety (CAS) score (r = 0.445, *p* < 0.05). In addition, the change of psychological well-being (PWBS) was negatively correlated with the change in COVID-19-related anxiety (CAS) (r = − 0.631, *P* < 0.01). Table 5 and Fig. 6 provides more details.

**Table 5:** Correlations between changes in the prevalence of low back pain, psychological well-being and Coronavirus-related anxiety

Variable	NMQ	PWBS
CAS	0.445*	− 0.631*

The data in the table is the correlation coefficient (r)

p* < 0.05; p** < 0.01

*CAS* Coronavirus anxiety scale, *NMQ* Nordic musculoskeletal questionnaire, *PWBS* Psychological well-being

## Discussion

This study aimed to evaluate the effectiveness of the Baduanjin intervention on the physical and mental health of college students during the COVID-19 pandemic. The research purpose was underlying the practical need to explore and find a harmless, non-drug intervention with minimal side effects in musculoskeletal and mental health problems caused by the COVID-19 pandemic and sedentary behavior. And further support the feasibility and acceptability of clinical trials of Baduanjin exercise. The main finding was that the reduction of COVID-19-related anxiety of college students is due to the 12-week training of Baduanjin. While no improvement was observed in the health education control group. In addition, participants in the Baduanjin group also showed a simultaneous decrease in the prevalence of lower back pain and psychological well-being. And the change of the prevalence of lower back pain and psychological well-being is significantly correlated with the improvement of COVID-19-related anxiety. Participants had no adverse reactions during the exercise intervention, and all participants were satisfied with the exercise program.

In our research, one of the most remarkable findings was that the effect of the Baduanjin intervention on COVID-19-related anxiety was significant and higher than that of the health education control group. After the intervention of the Baduanjin exercise, COVID-19-related anxiety has decreased significantly from 5.22 ± 0.45 to5.07 ± 0.27. The results are consistent with other studies [70–73] showing the Baduanjin exercise has a significant effect on alleviating anxiety symptoms. For example, Yu L et al. proposed that the Baduanjin exercise has a certain positive influence on COVID-19 patients in the Square cabin hospital, which is conducive to alleviating the anxiety and depression symptoms of the patients [72]. A systematic review concluded that the efficacy of Baduanjin exercise in reducing depression and anxiety symptoms in people with physical or mental illnesses [73]. In addition, some studies have shown that excessive exercise or exercise addiction harms anxiety and psychological well-being [74]. Simultaneously, during the pandemic, people who exercise more are less anxious than people who exercise less [75]. Therefore, exercise helps to relieve COVID-19 anxiety, fear, and stress.

Although the detailed mechanism by which Baduanjin improves COVID-19-related anxiety is not fully understood. As a comprehensive, multi-component intervention, Baduanjin may act through many intermediate variables along the pathway to improved anxiety outcomes. Several studies have suggested that COVID-19 is a new infectious disease with the characteristics of human-to-human transmission, long latency and high mortality [76–79]. There is still a lot of uncertainty about the origin, nature and process of the disease. Therefore, people are extremely lack of understanding of it. For the time being, there is no specific cure for the disease, which also aggravates people's panic and fear of COVID-19. Therefore, it is necessary to find suitable ways to alleviate people's anxiety and depression. These findings suggest that exercise-induced changes in the HPA axis modulate stress reactivity and anxiety in humans [80, 81]. Another possible mechanism for the anxiolytic effects of exercise is via mediation by the endogenous opioid system. Endogenous opioids have a role in the regulation of mood and emotional responses [82]. As a traditional qigong exercise, Baduanjin exercise has the advantage of accessible learning and no need for physical strength, relaxing the mind, and promoting sleep [83]. Some studies have also shown that Baduanjin exercise significantly reduces anxiety and depression [84–86]. These findings might reveal the likely neurobiological mechanisms of Baduanjin exercise in improving COVID-19-related anxiety of people.

Another result of our study found that with the improvement of COVID-19-related anxiety, the participants in the Baduanjin Group also showed significantly decrease in the prevalence of lower back pain and psychological well-being. Statistically significant changes were observed in these measures after the 12-week intervention. This improvement may be due to the benefits of regular Baduanjin exercise are expressed through adjusting breathing to make the process smoother, unifying mind and breathing, strengthening muscles and tendons to make the body more flexible, and the union of mind and body [87, 88]. At the same time, Baduanjin stresses ‘take the waist as the axis’ in practice [89]. Therefore, regular Baduanjin exercise enhances participants’ physical and mental health and decreases the prevalence of low back pain.

Several factors may explain the positive effects of Baduanjin training on the prevalence of lower back pain and psychological well-being in college students. First, the Baduanjin exercise can effectively decrease the prevalence of lower back pain. The present study is in keeping with these findings, since, as reported elsewhere, Baduanjin exercise can reduce lower back pain [43, 90]. And a significant positive correlation between the prevalence of lower back pain(NMQ)and COVID-19-related anxiety(CAS) (r = 0.445) was observed in the present study. Second, it found significant improvements in psychological well-being from Baduanjin training in the present study, related to the decrease of COVID-19-related anxiety of college students. This result is consistent with the research of other scholars [5, 91–94]. There was also a significant negative relationship between changes in COVID-19-related anxiety(CAS)and psychological well-being (PWBS) (r = − 0.631).

During the coronavirus pandemic, Baduanjin exercise can relieve stress and promote sleep quality, enhance mental health and well-being.

### Limited of the study

The function of Baduanjin during the COVID-19 pandemic has been mentioned many times above. Meanwhile, Baduanjin seemes to be a very interesting exercise for college students in different countries. It can be adapted to different needs by individualizing internal loadand intensity. It starts with a low dose and gradually increases itwhich makes it suitable for different groups.While this study has revealed many new angles for further research, there are limitations to the current findings.

First, This study has only speculated the possible mechanism of the Baduanjin exercise's influence on COVID-19-related anxiety, the prevalence of lower back pain, and psychological well-being through indirect indicators. Nevertheless, it did not explain in more detail the mechanism of the central nervous system and biological indicators.

Second, this study is limited by the impact of the pandemic, the lack of representativeness of research data and participants. Participants group consisted only on college students. Correlation studies should add different groups to strengthen and broaden the results, which is an important research direction in the future. Besides, outcomes of COVID-19-related anxiety and psychological well-being in this study were measured by subjective questionnaires (CAS and PWBS). Therefore, the subjective measures might have introduced a bias, leading to the potential overestimated intervention effects.

Third, the study did not mention the current pain level, whether the participants had chronic pain or whether participants took painkillers. It is worth considering in the future research.

Finally, the results show that the absence of follow-up beyond the 12-week Baduanjin exercise intervention period has had a beneficial effect on lower back pain, COVID-19-related anxiety, and psychological well-being. However, it is not sure whether the intervention effect still exists after 12 weeks.

## Conclusion

In short, this research shows that during the epidemic period, the 12-week Baduanjin exercise can alleviate the anxiety of college students about COVID-19, decrease the prevalence of low back pain, further promote the health of college students and enhance their psychological well-being. Besides, the Baduanjin is an effective, safe, and helpful exercise, which can improve different groups’ physical and mental health. In the future, more research should focus on the intervention means of the combination of non-drugs or the lowest dose of drugs that can bring health benefits to the different groups with sedentary lifestyle.

## Data Availability

The datasets analyzed during the current study are available from the corresponding author on reasonable request. And the data used for this study were part of a large international research project registered in the Protocol Registration and Results System. (ClinicalTrials.gov; https://clinicaltrials.gov/ct2/show/NCT04432038).
